# Easy-to-Build Textile Pressure Sensor

**DOI:** 10.3390/s18041190

**Published:** 2018-04-13

**Authors:** Francisco Pizarro, Piero Villavicencio, Daniel Yunge, Mauricio Rodríguez, Gabriel Hermosilla, Ariel Leiva

**Affiliations:** Pontificia Universidad Católica de Valparaíso, Escuela de Ingeniería Eléctrica, Avenida Brasil 2147, Valparaíso 2362804, Chile; piero.villavicencio.a@mail.pucv.cl (P.V.); daniel.yunge@pucv.cl (D.Y.); mauricio.rodriguez.g@pucv.cl (M.R.) gabriel.hermosilla@pucv.cl (G.H.); ariel.leiva@pucv.cl (A.L.)

**Keywords:** pressure measurement, textiles, wearable sensors, easy-to-build

## Abstract

This article presents the design, construction, and evaluation of an easy-to-build textile pressure resistive sensor created from low-cost conventional anti-static sheets and conductive woven fabrics. The sensor can be built quickly using standard household tools, and its thinness makes it especially suitable for wearable applications. Five sensors constructed under such conditions were evaluated, presenting a stable and linear characteristic in the range 1 to 70 kPa. The linear response was modeled and fitted for each sensor individually for comparison purposes, confirming a low variability due to the simple manufacturing process. Besides, the recovery times of the sensors were measured for pressures in the linear range, observing, for example, an average time of 1 s between the moment in which a pressure of 8 kPa was no longer applied, and the resistance variation at the 90% of its nominal value. Finally, we evaluated the proposed sensor design on a classroom application consisting of a smart glove that measured the pressure applied by each finger. From the evaluated characteristics, we concluded that the proposed design is suitable for didactic, healthcare and lifestyle applications in which the sensing of pressure variations, e.g., for activity assessment, is more valuable than accurate pressure sensing.

## 1. Introduction

Nowadays, wearable devices have become an important subject in the research community [1,2,3]. The possibility of connecting ordinary objects and obtaining data from them in real time has the potential to increase and improve the decision-making concerning a number of objects and situations that could not previously be measured. This opens up a substantial new area of applications in smart industries, as well as in the domains of education, biomedicine, and healthcare [4,5,6,7,8,9].

In order to turn these ordinary objects into candidates for wearable connected devices, it is important to make the sensors and communication systems as lightweight, flexible, and thin as possible, so that they use a low volume when integrated into a system [10]. It is under these conditions that textile sensors, which introduce the smart clothing concept, have been an interesting topic in recent years, based on the fact that clothing and apparel have large portable surfaces into which sensors and electronics can be integrated [11,12,13,14].

Several studies on the development of textile sensors and its physical characteristics have been conducted in the last years [15,16,17]. These studies include flexible temperature sensors [11,18], strain gauge sensors [15,19,20], electromagnetic induced sensors [21] and pressure sensors [22]. Amongst these sensors, pressure sensors have been widely studied because of its multiple applications, such as in the healthcare domain [23,24,25,26,27], sports monitoring [28,29,30] and eating habits monitoring [31].

Specifically, pressure sensors have different ways of measuring the pressure variation [32]. These sensors can work either measuring capacitive changes [24,29,33], piezoelectric voltages generated by pressure [34], or resistance variation generated by pressure [31], where piezo-electrical [35] and piezo-resistive [36] polymers are widely used for these purposes. 

One common characteristic of these pressure sensors is that they necessitate the use of machinery, for example sewing machines, in order to be built. This characteristic limits the possibility of replicating sensors for educational purposes, where machinery is either expensive, or difficult to obtain. In order to introduce wearable technologies in engineering schools, it is important to students to be able to deal with the implementation aspects of such sensory systems, and to evaluate independently the issues and advantages of textile sensors in a real-world scenario. Therefore, it is necessary to have easy-to-build textile sensors in order to permit students to understand the sensing and data interpretation processes involved.

This article presents the design, construction and evaluation of an easy-to-build textile resistive pressure sensor. The sensor was evaluated in terms of linear range, construction repeatability and recovery time. Finally, the sensor was used in a classroom environment application.

## 2. Sensor Design and Construction

**Figure 1:**
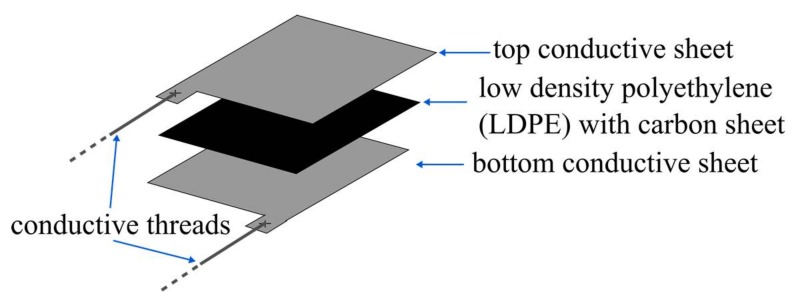
Exploded view of the resistive textile pressure sensor.

**Figure 2:**
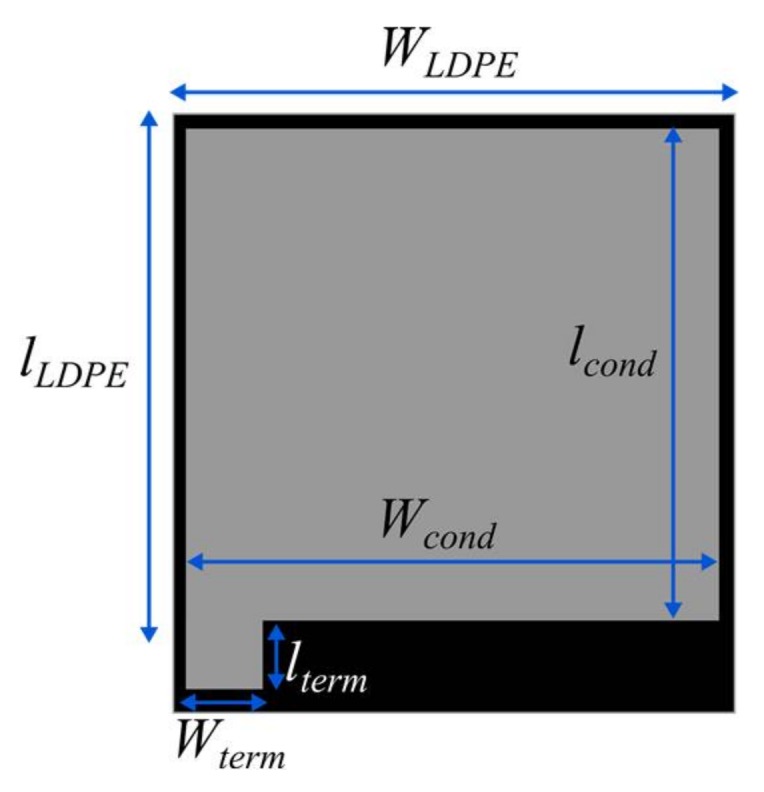
Top view with dimensions of the resistive textile pressure sensor.

The designed sensor works on the basis of the resistance variation through pressure application. The sensor itself is based on a structure proposed in [36] and consists of a sandwich made of two conductive Shieldex NoraDell woven fabric sheets [37] with a Low Density Polyethylene (LDPE) sheet between the conductive fabrics, which makes it different from previous designs in literature [36]. The Shieldex NoraDell woven fabric sheets have been largely used for microwave applications due to its good conductivity and radiation efficiency characteristics [38,39,40,41], having a surface resistance lower than 0.009 Ω/sq and a maximum thickness of 0.13 mm. Between the two conductive sheets is placed a sheet of Antistat black conductive bag (ANT006BCB) [42] widely used for anti-static applications. These bags are made of Low Density Polyethylene (LDPE) with a 0.1016 mm carbon layer, having a surface resistivity of 10^4^ to 10^6^ Ω. The combination of the conductive textile sheets with the polymer sheet creates a piezo-resistive effect that translates the applied pressure on the sheet sandwich into a resistance variation [36]. Finally, the conductive sheets are connected through Shieldex 117/17 DTEX conductive threads for the measurement of their resistance. Figure 1 shows an exploded view of the designed sensor. In terms of the geometry of the sensor, and in order to make an easy and replicable design, a square-shaped sensor was constructed. A top view of the sensor is shown in Figure 2.

**Figure 3:**
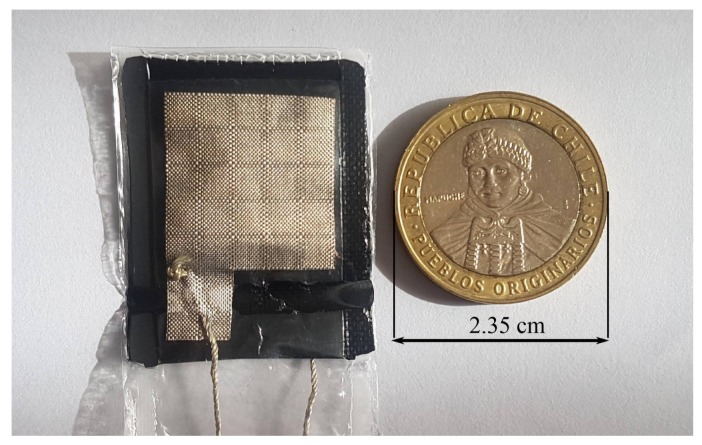
Top view with dimensions of the resistive textile pressure sensor.

The conductive textile sheet has a surface of $W_{cond}\times l_{cond}$ where $W_{cond}=l_{cond}=2$ cm. The conductive textile sheet has also a small surface of $W_{term}\times l_{term}$ that acts as a terminal for the sensor and where the conductive thread is sewed. The dimensions of the terminal are $W_{term}=0.7$ cm and $l_{term}=0.6$ cm. Regarding the LDPE sheet, the surface $W_{LDPE}\times l_{LDPE}$ is larger than the conductive textile sheets in order to avoid direct electrical contact between them. The dimensions of the LDPE sheet are $W_{LDPE}=2.5$ cm and $l_{LDPE}=3.3$ cm. Finally, the three sheets are glued together with a thin double-sided adhesive tape, and sealed with a flexible plastic film for protection. The final textile resistive sensor is depicted in Figure 3.

The construction of the sensor is relatively easy, and was done using only standard office tools and done by hand. The construction procedure, which consists of measuring and marking the dimensions on each sheet (conductive and LDPE), cutting and consolidating the sensor, does not take more than 15 min for a person who reads the building instructions for the first time. This characteristic makes the sensor suitable for its use in a classroom environment.

## 3. Measurement Results

In order to validate the pressure sensor, several measurements were done. Five different scenarios took place to validate different aspects of the constructed sensor, namely time response, linearity and replicability. For the time response, the recovery time when the sensor was released was assessed. Then, for the linearity evaluation (hysteresis and variance), the sensor was measured when decreasing and increasing pressures were applied. Finally, all these measurements were repeated over five constructed sensors in order to validate their replicability.

### 3.1. Recovery Time

The first important variable to measure for wearable sensors is the recovery time. This is the time that the sensor requires for getting back to its nominal state or zero-state once the pressure is no longer applied. The measurement of this time must be performed first, because it determines the minimal and maximal waiting times between the subsequent measurements. 

For this measurement, the constructed sensor is connected to an Arduino LilyPad, where the resistance variation as a function of time is measured through the LilyPad with an application written for that purpose. To apply the pressure to the sensor, a special container was constructed in order to put weights and apply a controlled and uniform pressure over the sensor. The container was constructed using a 3D printer, fed with standard polylactic acid (PLA) filaments. The base dimensions of the container were defined the same as those of the conductive sheet of the sensor ($W_{cond}\times l_{cond}$) in order to apply a uniform pressure over the sensor.

**Figure 4:**
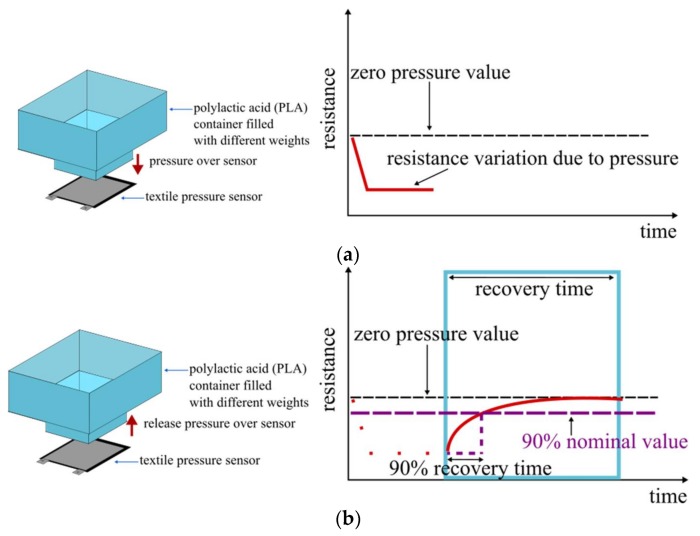
Measurement of recovery time of the textile pressure sensor: (**a**) Applied pressure to the sensor and resistance-time variation. (**b**) Released pressure from the sensor and resistance-time variation.

Finally, in order to measure the recovery time, an offset was defined as the zero value of the sensor. This offset was set at 2.4 kΩ which corresponds to the average measured resistance when there is no pressure applied over the sensor. To measure the recovery time, a pressure is applied to the sensor. Once the sensor reaches the resistance value that corresponds to the applied pressure, the pressure is taken away from the sensor. The recovery time corresponds then to the time from which the pressure is no longer applied over the sensor, till the time at which the sensor reaches the defined offset. Another time that will be measured will be the time to the sensor for come back to a 90% of the nominal value. This value can be useful, for example, for dynamic pressure sensing applications. Figure 4 shows the measurement procedure of the recovery time.

For the test, the applied pressure varies from 2 to 70 kPa. Table 1 shows the average measured values of the recovery time of the constructed sensors for each applied pressure and the average measured values of recovery time to a 90% of the threshold.

The measurement results show that the maximum recovery time was obtained with an applied pressure of 50 kPa and the lower time was obtained at 2 kPa. There is no noticeable tendency towards a time variation with the augmentation of the pressure. The recovery time was found between 30 and 35 s for pressures above 6 kPa. For the comeback time to the 90% of the defined threshold, it was found that for lower pressure (from 2 to 8 kPa) the sensor almost instantly comes to a resistance value that is within the established threshold. For higher pressures, the times varies from 13 to 17 s, depending on the applied pressure. 

### 3.2. Decreasing Pressure Range

To evaluate the hysteresis aspect we separately evaluate the sensors when decreasing and increasing pressure is applied to them. For the decreasing pressure test, the resistance value is measured between the terminals of the sensor as a function of a time-decreasing and equally applied pressure over the surface of the sensor. Two variants of the test were conducted; one having a short pause between measurements (30 s), and the other having a long pause between measurements (180 s). This was done to check for time response effects.

**Figure 5:**
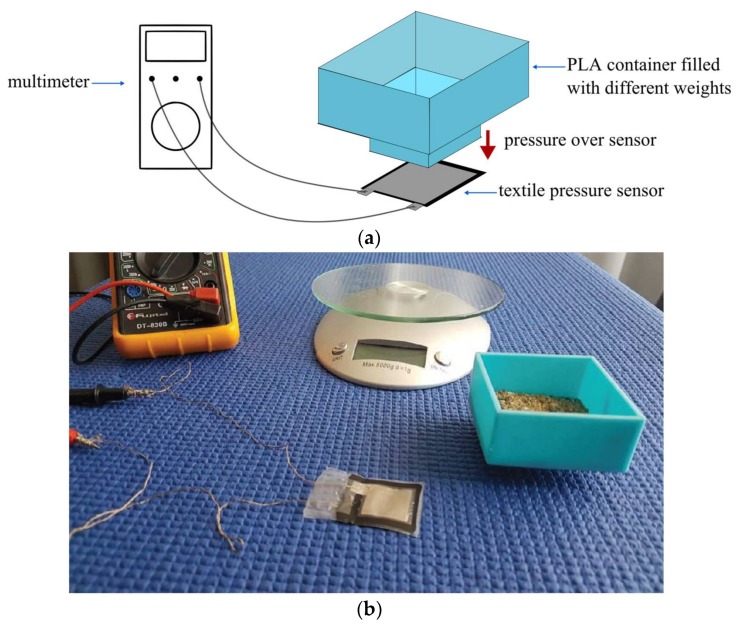
Measurement setup used for pressure variation measurement: (**a**) Schematic of the measurement setup. (**b**) Picture of the measurement setup.

The measurement setup is shown in Figure 5a. For this measurement, the container is filled with the desired weight and placed over the pressure sensor to measure the resistance variation using a multimeter connected to the textile conductor. Figure 5b shows a picture of the implemented measurement setup.

**Figure 6:**
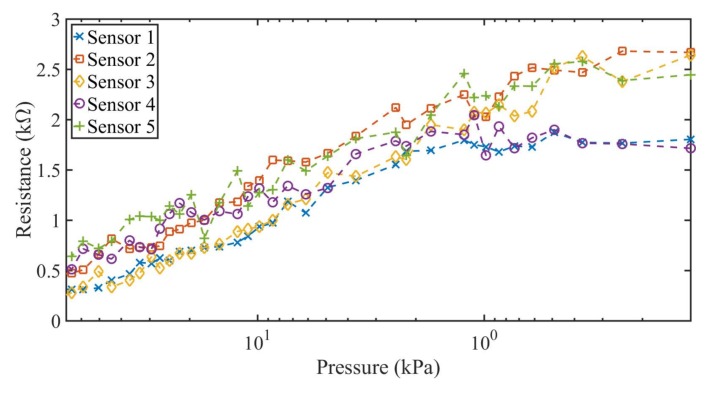
Measured resistance against decreasing pressure variations for the five constructed textile pressure sensor with short pause time (30 s) between measurements.

**Figure 7:**
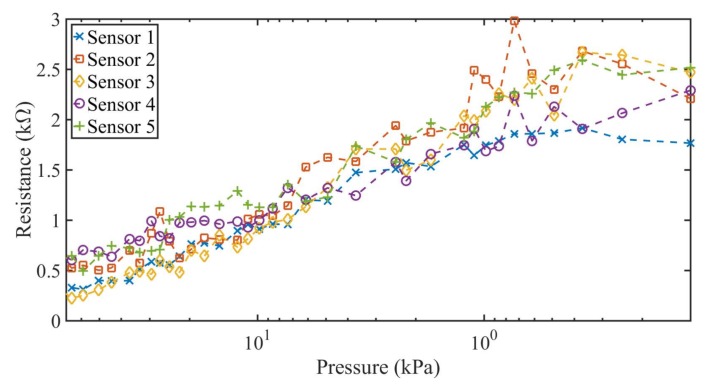
Measured resistance against decreasing pressure variations for the five constructed textile pressure sensor with a long pause time (180 s) between measurements.

The measurement results of both case scenarios are shown in Figure 6 and Figure 7 for the five constructed sensors. The applied pressure varies from 70 kPa to 0 kPa with a pause between tests of 30 s and 180 s, respectively. From the results shown in Figure 6 and Figure 7, two characteristic zones of the sensor can be determined. At the lower applied pressures (from 0 to 1 kPa), there is no significant variation of the resistance of the sensor, as expected. For this reason, sensors in the literature [27,29,43] do not use this pressure range, especially when movement or folding are involved [27]. For pressures above 1 kPa, a wide linear range of resistance variation was obtained for the five constructed sensors. The average resistance in this zone varied from 2.2–0.41 kΩ for applied pressures between 1 kPa and 70 kPa.

### 3.3. Increasing Pressure Rrange

**Figure 8:**
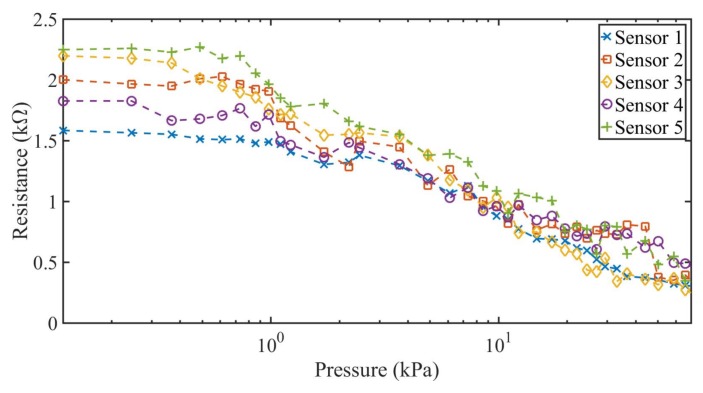
Measured resistance against increasing pressure variations for the five constructed textile pressure sensor with short pause time between measurements.

**Figure 9:**
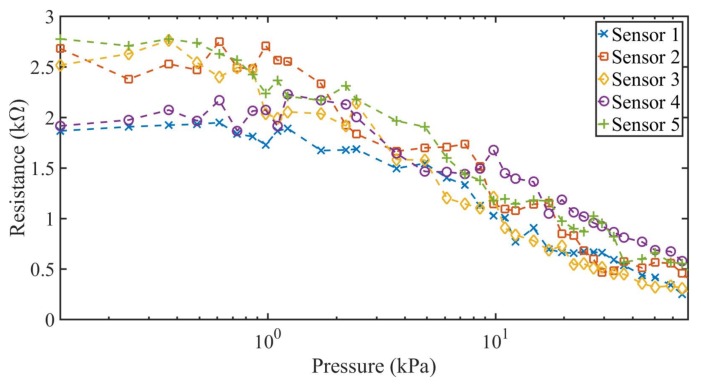
Measured resistance against increasing pressure variations for the five constructed textile pressure sensor with long pause time between measurements.

The same previous scenarios (short pause and long pause between measurements) were used, this time with increasing values of pressure applied over the textile pressure sensor. Figure 8 and Figure 9 show the results of the resistance variation as function of an increasing applied pressure with 30 s pause (short time) and 180 s pause (long time) between measurements. The measurement results are comparable to the results obtained with the decreasing pressure against resistance in terms of linear range and resistance variation, having the same linear variation of the resistance for the five constructed sensors between 1 kPa and 70 kPa. For the linear zone of each individual sensor, it can be observed that there is no significant hysteresis effect.

### 3.4. Sensor Comparison and Model

It is important to compare and model the constructed sensors in order to determine their substantial differences at the time of constructing this kind of sensor. In order to compare the sensors, a model of the average resistance obtained in function of the applied pressure to each sensor was developed. For this model, only the linear part of the sensor was taken into account, comprising pressures from 1–70 kPa. A logarithmic fitting was obtained from the measured data for each sensor, described in the following equation:

(1) R=b+m⋅log(P)

where *R* is the resulting resistance in kΩ , *P* is the applied pressure in kPa, *m* the slope and *b* the intercept of the linear function. 

As the individual behavior of each sensor is very similar in both cases of increasing and decreasing pressures, the linear function of each sensor was generated from the data of both tests. Furthermore, a single fitting was done for all sensors using all the available measurement data. Table 2 shows the intercept, slope and Mean Square Error (MSE) using the fitting equation in (1), one for each sensor individually, and one for all the measurements done for all sensors using a single fitting generated from all the available data.

**Figure 10:**
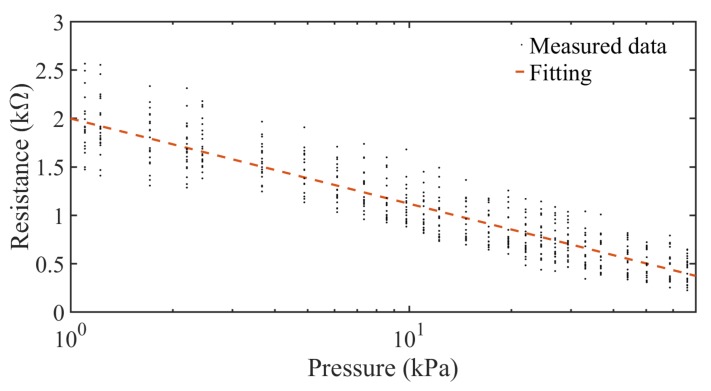
Measured data of the five constructed sensors and logarithmic fitting of the data.

As shown in Table 2, all the sensors have the same decreasing linear behavior in terms of resistance over the augmentation of the pressure, with negligible differences in the slope and intercepts values of the function. Furthermore, the maximum MSE for the constructed sensors is 0.04, which matches the MSE obtained taking into account all the measurements obtained from the five constructed sensors collectively, as shown in Figure 10. The difference found in the intercept and the slope among sensors can be associated with the lax construction tolerances of each sensor, which are acceptable for a sensor completely constructed by hand. On Table 3 are shown the dimensions of each constructed sensor measured with a precision ruler.

## 4. Use-Case of the Proposed Sensor

A use-case using the proposed sensor was done in a classroom environment, where the students construct and measure the sensor inserted in an application. The characterization of the sensor shows that the sensor has a linear behavior when pressures above 1 kPa are applied, with a mean recovery time of 30 s. These characteristics, adding the fact that the sensor has a fabrication tolerance reflected on the MSE, make the sensor difficult for its use in static pressure measurement for critical safety applications, where a threshold of pressure is determined in order to make an action. Nevertheless, the sensor characteristics can be suitable for applications where it is important to measure variations or tendencies of applied pressures as for example, lifestyle applications (e.g., posture correction, walking behavior).

The proposed application for the proof of concept consists in a glove where the pressure sensors are installed on its fingertips and measure the behavior of the sensor when a pressure is applied (e.g., when grabbing and holding a glass). The objective of this application is to recognize when the glove is holding an object, and which finger makes the higher pressure to hold it.

**Figure 11:**
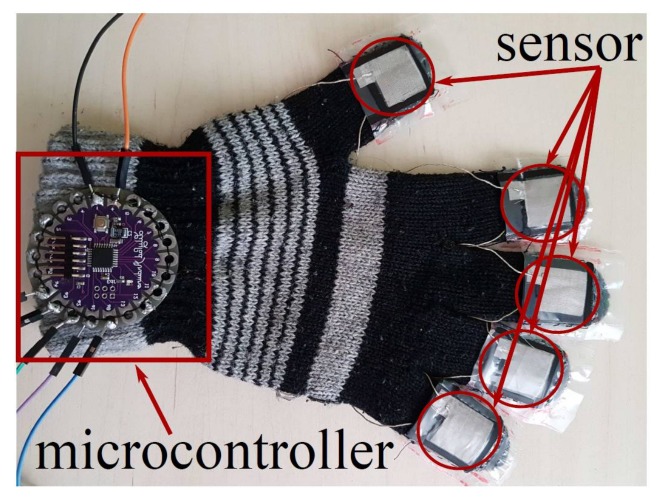
Wool glove with pressure sensors installed and connected to microcontroller.

**Figure 12:**
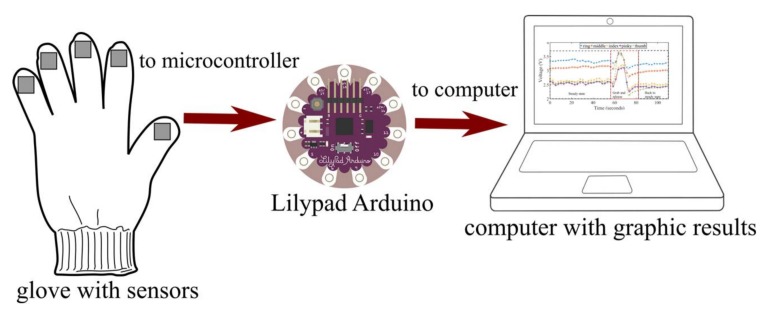
Experimental setup for pressure variation measurement using proposed easy-to-build sensor.

First of all, the students construct and install the sensors in a wool glove using an instructive given to them. Figure 11 shows the sensors installed on the glove. The students connect the sensors to an Arduino Lilypad (state of the art wearable microcontroller). The Lilypad measures the pressure variation through a voltage difference caused by the resistance variation of the constructed pressure sensors. This was done using a dedicated application installed by the students. Finally, the microcontroller was connected to a computer for visualization of the results. Figure 12 shows the experimental setup done by the students.

**Figure 13:**
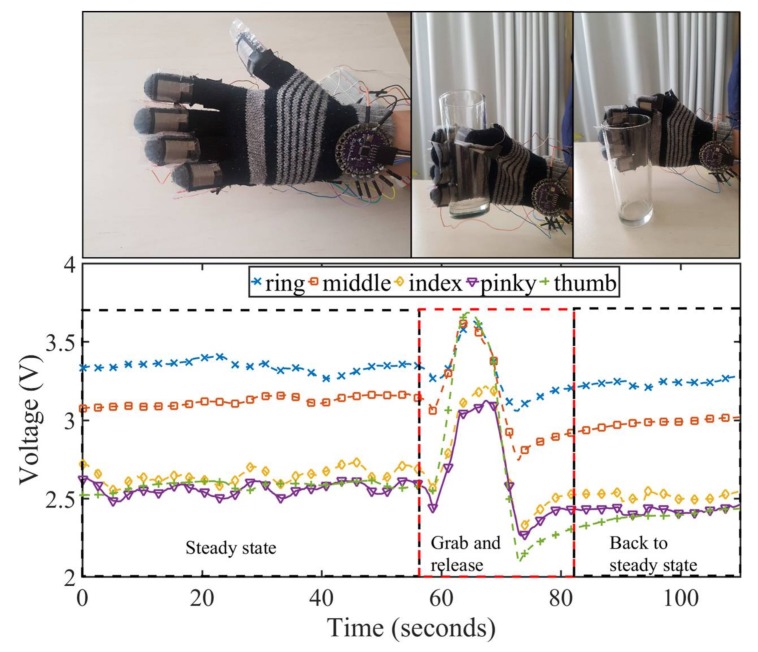
Measured voltage data of five constructed sensors placed in the fingertips of a glove holding a glass.

Figure 13 shows the measurement results obtained with the sensors installed on the glove on each fingertip. The measured experience consisted of three steps: the glove in steady state, the glove grabbing and holding an object and finally releasing the object. First of all, we can notice that there is a slight difference in the steady states amongst the sensors. This can be explained due to the constructed tolerance of each sensor. Another factor that might influence the steady state resistance of each sensor is that the different curvatures of the fingertips deform the sensors differently.

The second noticeable characteristic of the measurement is when a glass is grabbed using the glove. It can be clearly noticed the change of the pressure applied by each individual finger translated on the voltage lecture on the microcontroller. For example, we can notice that the thumb is the finger that applies more pressure when grabbing and object. Finally, when the glove releases the glass, we can notice the release behavior on the sensors and clearly the tendency to come back to the steady state. Another remarkable aspect of this measurement is that the recovery time can be redefined as thresholds within a percentage of the initial value, as now dynamics are measured. Under these terms, the recovery time can be drastically reduced. In this use-case, as shown in Figure 13, if a threshold of the 90% of the initial value is set, the recovery time is reduced to 5 s.

With this experiment the students have accomplished the whole system comprehension in terms of sensor construction, connection, programming and interpretation of measured results to characterize the behavior of each finger when holding a glass.

## 5. Conclusions

An easy-to-build textile pressure sensor was designed, constructed and evaluated. The sensor consists of two sheets of conductive woven tissue that surrounds a conventional antistatic black conductive sheet. Five measurement scenarios where considered: increasing applied pressure with short time release between measurements, increasing applied pressure with longer time release between measurements, decreasing applied pressure with a short time release between measurements, decreasing applied pressure with a long time release between measurements, and the release time or recovery time of the sensor. The recovery time was measured, obtaining average times that varied from 22 to 35 s depending on the applied pressure, and between zero and 17 s if we consider the 90% of the threshold, also depending on the applied pressure. To notice that in this case, for pressures below 10 kPa, the comeback time to the 90% of the threshold is negligible. In addition, as a possibility for improving the recovery time, we propose the assessment of different anti-static shields and adhesion methods between sheets that may have different elastic properties. 

The constructed sensors showed two distinctive zones in terms of resistance variation as function of the applied pressure. The first zone between 0 and 1 kPa showed no significant variation of the resistance as the pressure increased. This can be expected as the pressures applied were low, and textile pressure sensors hardly work on that range of pressure in the literature. The other resistive zone of the sensor presented a linear behavior within pressure values from 1 to 70 kPa. These applied pressures corresponded to an average variation of the resistance of 2.2 kΩ to 0.41 kΩ. Finally, a fitting equation for each sensor individually and for all sensors collectively was obtained. All the curves obtained presented the same linear behavior, with a comparable MSE, and slight differences in the intercept and the slope. These differences can be associated with the construction discrepancies of each sensor, as expected on a handmade constructed sensor. These results make the sensor more suitable for dynamic pressure sensing applications, where the tendency of the signal is the valuable data to acquire. Moreover, the sensor can be evaluated with uneven pressures applied to it in order to extend the number of applications.

Finally, and taking into account the sensor characteristics, a proof of concept was done in a classroom environment, using the proposed sensor installed in the fingertips of a glove, to measure the pressure variation of each finger when holding a glass with the glove. The measurement results shows that the sensor is useful for this kind of applications, where it can be easily differentiate the steady-state and the impulse response of the system, translated into pressure variations. 

The obtained results are promising in terms of possible applications. The advantage of this sensor is that it can be built using classical and basic tools without using any machine and in a period which does not exceed the 15 min for a first-time constructor of the sensor. The easy-to-build characteristic makes the sensor suitable for pedagogic applications, considering that nowadays, wearable devices and Internet of Thing (IoT) are trending topics in research. This sensor will be helpful to teach new engineers important aspects of wearable and connected devices. On this work, for example, an Arduino LilyPad application was written to measure the recovery time, which can be easily extended to communicating the sensing data. Future works include the evaluation of the proposed sensor on a robotic arm, for medical telemonitoring, and structural health monitoring applications.

## Figures and Tables

**Table 1:** Average recovery time of the constructed sensors as function of the applied pressure.

Applied Pressure (kPa)	Recovery Time (s)	90% Recovery (s)
2	22.6	0
4	24.6	0
6	30.4	0
8	28.9	1
10	30.0	1.9
20	32.0	15
30	36.0	15
40	30.6	14.3
50	35.3	13
60	32.3	14
70	35.3	17

**Table 2:** Fitting parameters and Mean Square Error obtained from each individual sensor and from all the sensors collectively.

	Intercept	Slope	MSE
Sensor 1	1.964	−0.826	0.01
Sensor 2	2.175	−0.978	0.04
Sensor 3	2.002	−1.002	0.01
Sensor 4	1.877	−0.700	0.03
Sensor 5	2.148	−0.889	0.02
All sensors	1.997	−0.881	0.04

**Table 3:** Conductive sheet dimensions (in centimeters) of each conductive sensor.

	Sensor 1	Sensor 2	Sensor 3	Sensor 4	Sensor 5
$W_{cond}$	1.991	1.85	1.92	1.91	1.91
$l_{cond}$	1.8	1.89	1.81	1.86	1.82
